# Toxicological Analysis of Illicit Drugs Seized in Naples (Italy) and First Detection of Synthetic Cannabinoids during COVID-19 Pandemic

**DOI:** 10.3390/healthcare10081488

**Published:** 2022-08-08

**Authors:** Anna Carfora, Raffaella Petrella, Giusy Ambrosio, Ilaria Fracassi, Stefano Festinese, Alessandro Feola, Carlo Pietro Campobasso

**Affiliations:** Forensic Toxicology Unit, Department of Experimental Medicine, University of Campania “Luigi Vanvitelli”, Via Luciano Armanni, 80138 Naples, Italy

**Keywords:** synthetic cannabinoids, new psychoactive substances, illicit drugs in seizures, COVID-19 pandemic, cannabis

## Abstract

The COVID-19 pandemic has consistently raised the number of drug seizures, in United States as in Europe. The COVID-19 pandemic has also changed the typology of seizures from “more traditional” drugs to New Psychoactive Substances (NPSs), depending on geographical area. In Europe, the most frequent NPSs are synthetic cannabinoids (SCs) and cathinones, nonetheless synthetic opioids and phenethylamines are widely used. The aim of the study is the detection of NPS and other substances of abuse available in the black market, by quali/quantitative methods in LC-MS/MS and GC-MS. From 2018 to 2021, 268 seizures occurred in total and were analyzed by the Forensic Toxicology Laboratory (FTL) of Naples (Italy). The distribution of analyzed seizures over the years is the following: 53 in 2018, 61 in 2019, 89 in 2020 and 65 during the first semester of 2021. Cannabis was the most detected drug both in hashish and marijuana seizures, followed by cocaine > heroine > prescribed drugs > ketamine–amphetamine MDMA. No NPSs were seized until June 2021, when NPSs were found in two different seizures: Case #1 showed a bar of Cannabis resin containing a low level of Δ^9^THC = 0.57% associated to SC AB-FUBINACA in 4.5%; Case #2 showed a vegetal resinous substance (Δ^9^THC = 0.27%) with SC 5F-APINACA (4.1%) associated with methadone (1.0%). The detection of NPSs is alarming evidence that can lead to an increase in the risk of overdose or other negative and unpredictable consequences, such as violent or self-harming behavior in unaware users of cannabis derivatives considered of “*natural*” origin.

## 1. Introduction

New Psychoactive Substances (NPSs) are defined by the United Nations Office on Drugs and Crime (UNODC) as “substances of abuse, either in a pure form or a preparation, that are not controlled by the 1961 Single Convention on Narcotic Drugs or the 1971 Convention on Psychotropic Substances, but which may pose a public health threat” [1]. NPSs are a complex group of substances often known as synthetic drugs or, in the more popular slang, as “legal highs” [2]. In Europe, the most frequent NPSs are synthetic cannabinoids (SCs) and cathinones; nonetheless, synthetic opioids and phenethylamines are widely used. According to the European Monitoring Centre for Drugs and Drugs Addiction (EMCDDA), approximately 64% of the seized SCs was in the form of herbal mixtures and 13% in the form of processed powders in “smokable herbal mixtures” [3,4]. Based on epidemiological studies and survey data from recreational consumers, SCs can be easily bought in the form of powder, oily paste, herbal mixture, or incense [2]. They can also be synthetized as resins. SCs are the third most popular drug class among the adolescents after marijuana and amphetamines [5]. The growing spread of NPS is a real challenge in forensic toxicology. Typically mimicking the effects of classical controlled drugs such as amphetamine, cocaine, cannabis, heroin, and LSD, NPSs have often utilized various well-established templates (e.g., phenethylamine, piperazine, aminoindan, cathinone), modified to either bypass domestic or international drug legislation or to boost their effects, often resulting in increased harm to users. This is the reason why, in some countries, various methods of drug control have been introduced [6]. However, several substances that mimic the controlled drugs are still unscheduled, unregulated, and not under the auspices of international law. Modification of abandoned drugs or new entities with slight or major structural variations can transform the restricted progenitor drug into an uncertain category of legal status. This gray legal status is an incentive for entrepreneurs to introduce new drugs quickly into the global market [6,7].

In US, the alarming epidemic of drug-related fatalities observed in the past few years has been also referred to the abuse of SCs. More recently identified cannabinoids include XLR11, AB-FUBINACA, and AB-PINACA [5]. Distinctly different from the progenitor, they are a conglomerate of compounds designed to mimic the effects of THC in marijuana, and so by targeting the cannabinoids receptors in brain. However, these SCs can activate some metabolites showing more robust and persistent effects, and other non-cannabinoid brain receptors. These drugs are involved in numerous medical emergencies and reports of acute toxicity.

In February 2010, symptoms such as agitation, hallucinations, mental confusion, panic attacks, tachycardia, and cerebral insufficiency were reported in Northern Italy from the Italian Emergency Departments, in which six cases of acute intoxication by the “JWH-018”, an SC better known as “n-Joy”, were diagnosed. In 2010–2011, the National Early Warning System (NEWS) of the National Institute of Health (NIH) has reported, in total, 11 cases of acute intoxication by “JWH-018” [8]. In 2010, the presence on the black market of another SC, such as “JWH-122”, sold with multiple slang names such as “Jungle Mistic Incence”, “Spice”, “Bonzai”, “Maya”, has been also reported in Northern Italy. The SC “JWH-122” has been also found in a seizure in the Molise Region (Southern Italy) in 2011 [8]. NPS are not that common in Italy, such as in other European Countries [9]. Data from the EMCDDA report 2016 show that “in the overall structure of illicit drug seizures, cannabis products dominate, followed by cocaine and heroin, with other substances (mainly synthetic stimulants) seized less frequently” [10]. According to the EMCDDA report from 2017, NPS were found in 30 seizures, all coming from the Northern or Central Italy [3,11]. In 2020, the NEWS of the NIH identified 128 NPS, among which only 38 substances were already known in Europe. In Italy, NPSs seem to be more frequent in Northern Italy compared to Southern and Central Italy [12], where a prevalence of marijuana and hashish, followed by cocaine and heroin, has been observed [9]. Among the NPS, synthetic cathinones were the most reported substances (28%) in Italy from 2016 to 2021, followed by cannabinoids (14%), opioids (12%), and arylcyclohexylamines (12%) [12]. In 2014 and 2015, an outbreak of NPS in seized drugs has been reported in Florence, represented mostly by synthetic cathinones, phenethylamines, and AB-FUBINACA [13]. Similar findings were observed in 2014 in Modena and Reggio Emilia by Verri et al. (2019) [14]. Both studies showed that cannabis was the most-seized drug, followed by cocaine in large cities of Northern Italy.

In this study, toxicological analyses on illicit drugs seizures were summarized in order to define the observed trend at local level and the more common drugs of abuse. The Forensic Toxicology Laboratory (FTL) of the University of Campania “L. Vanvitelli” (Naples, Italy) is regarded as “Collaborative Centre of NEWS”. The FTL periodically receives NPS standards in order to develop innovative methods in LC-MS/MS and GC-MS. It can detect the presence of new substances or other traditional substances available on the black market, in the short term and with highly specific methods in non-biological findings. Illicit drug seizures were provided by the police in cases of trafficking or of possession for personal use from 2018 to the first semester of 2021.

## 2. Materials and Methods

### 2.1. Sample Collection and Preparation

For each seizure, a representative sample was collected for qualitative and quantitative analysis according to procedures described in the literature [15]. General screening of drugs was performed by using analytical instrumentations such as gas chromatography coupled with mass selective detector (GC–MS), and liquid chromatography–mass spectrometry triple quadrupole (LC-MS/MS). A suitable amount of solid sample (about 30 mg for powder/solid/tablets and about 100 mg for dry or resinous vegetal substance) was dissolved in ethanol, and in case of liquid sample, 1 mL was used with appropriate sample preparation before reconstitution in ethanol [16,17].

### 2.2. GC-MS Analysis

For analysis of drugs in seized materials using GC–MS, an Agilent 7890A gas chromatograph coupled with a 5977C mass selective detector (Agilent Technologies, Santa Clara, CA, USA) was used. The GC was equipped with a HP-5MS capillary column (30 m, 0.25 mm, 0.25 m) and the helium flow rate was set at 1.0 mL/min. A total of 1 L of sample solution was injected into the GC for analysis. The typical temperature settings of GC were as follows: injector temperature, 270 °C; initial column temperature, 70 °C; hold time, 1.0 min; temperature ramp, 30 °C/min–180 °C, then 7 °C/min to 300 °C, final temperature, 300 °C; hold time, 10.0 min. The mass selective detector in a full scan mode in the range of *m/z* 40–450 was operated in electron ionization mode with the electron beam energy set at 70 eV.

### 2.3. LC-MS/MS Analysis

Liquid chromatography mass spectrometry-based methods offer more sensitive and specific identification and quantification for each individual NPS. An HPLC Agilent 1200 equipped with a column Zorbax 08-C18 (4.6 mm × 50 mm, particle size 1.8 m), coupled with an AB-Sciex API 3200 mass spectrometer; Scan type: MRM; Polarity: Positive; scan time: 0.01 s; Scan width: 0.2 *m/z*; Eluent: sol. A: H_2_O + 0.5% Formic Acid and Sol. B: Acetonitrile (AcN) + 0.5% Formic Acid. The separation was carried out with the use of 0.01 M ammonium formate (pH 3) (A) and acetonitrile (B) mixture as mobile phase at a flow rate of 0.9 mL/min and using a gradient elution program: A:B = 90:10–20:80 for 30 min and hold for 5 min. The injection volume was 5 μL.

## 3. Results

### 3.1. Drug Seizures Distribution

**Figure 1:**
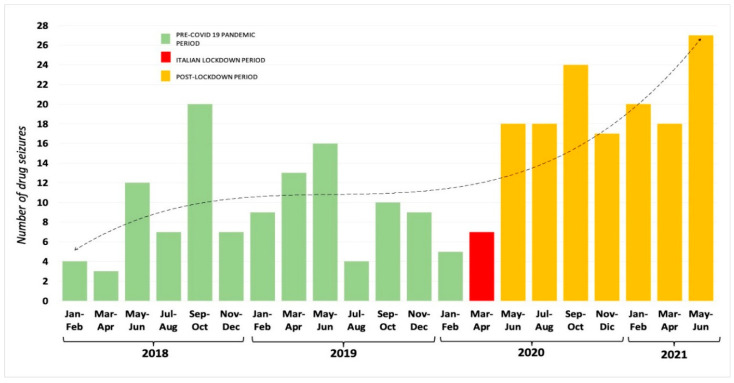
The distribution of seizures occurred in Naples (Italy) from January 2018 to June 2021, comparing the pre-pandemic period (green), with the lockdown (red) and the post-lockdown periods (yellow). The dotted line represents the trend obtained by the polynomial function.

From 2018 to 2021, 268 seizures in total, which occurred in Naples, have been analyzed by FTL. The distribution of seizures over the years is as follows: 53 in 2018, 61 in 2019, 89 in 2020, and 65 during the first semester of 2021. The distribution of drug seizures through the pre- and ongoing pandemic years (2018–first semester of 2021) shows a decrease of seizures (12 seizures) during the first wave of the pandemic, (from January to March 2020), followed by an increase (36 seizures in May–July 2020) in the remaining months of 2020 that exceeded the values of the pre-COVID-19 period (Figure 1). The first semester of 2021 showed also a remarkable increase of the number of seizures (65 in total) compared to the pre-pandemic period, with only 61 cases reported in 2019.

### 3.2. Drug Seizures Typology

The typology of seized drugs from the beginning of 2018 up to June 2021 is summarized in Table 1. Cannabis is the mainly detected drug, both in hashish and marijuana seizures. The other drugs found in the 268 seizures are the following: cannabis > cocaine > heroine > prescribed drugs > ketamine–amphetamine/MDMA. No NPSs have been found except for two seizures that occurred in the first semester of 2021. During the pandemic period, a relevant change in drug use has been observed, in both quantitative and qualitative ways. After a comparison of the most common drugs found in seizures between the pre-pandemic period and the post–lockdown one, a noteworthy increase of cannabis derivatives on the black market has been found: in the pre-pandemic period from 2018 to 2019, 64 Hashish and Marijuana seizures were reported in total compared to 93 Hashish and Marijuana seizures from 2020 to June 2021. It is worth mentioning that in the first semester of 2021, 65 seizures in total occurred. Such a large number of seizures has overcome the total number of seizures occurred in 2019 (61 cases), with cannabis by-products in the lead (38/65 cases).

### 3.3. NPS Detection

**Figure 2:**
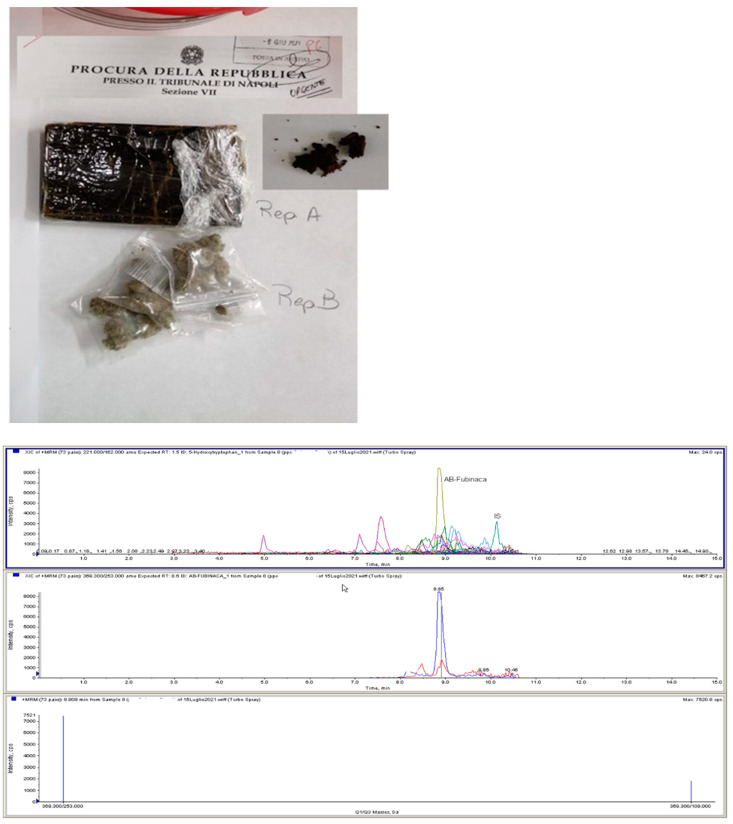
Case #1: a bar of cannabis resin (Rep. A) containing a low level of Δ^9^THC (0.57%) and the synthetic cannabinoid AB-FUBINACA (graphic in LC-MS/MS) in a 4.5% concentration. In the same seizure, a second small package was found (Rep. B) containing cannabis inflorescences with 12.75% Δ^9^THC concentration.

In June 2021, for the first time in Naples, NPSs were found in two different seizures. In both cases, results showed cannabis derivatives, differently packaged. In the first seizure (Case#1, Figure 2) there were two different packages: the first package was a bar of cannabis resin (Rep A) containing a low level of principle (Δ^9^THC = 0.57%) but also the synthetic cannabinoid AB-FUBINACA in a 4.5% concentration. In the second package, cannabis inflorescences (Rep B) were found with 12.75% Δ^9^THC concentration.

**Figure 3:**
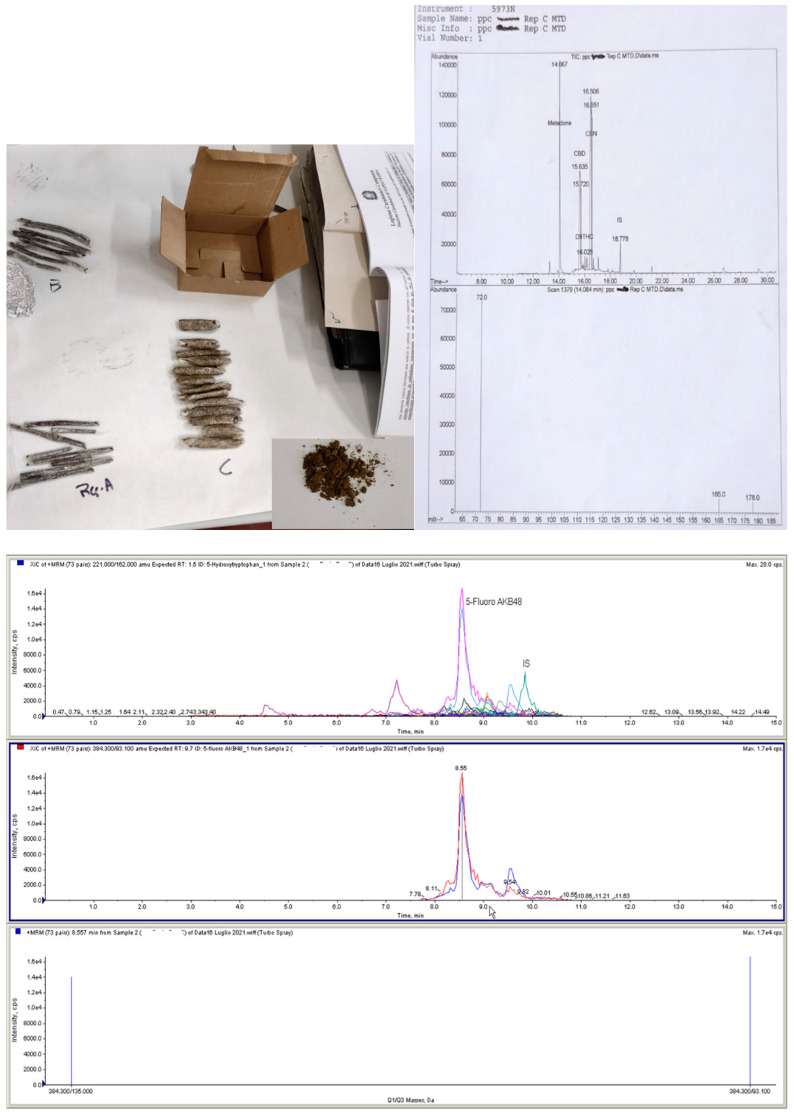
Case #2: the long thin bars (Rep A and B) resulted in Hashish with 11.85% and 10.75% Δ^9^THC concentrations, respectively. In the small fat cigars (Rep. C) containing a brown vegetal resinous substance showed the presence of Δ^9^THC = 0.27% (level of active principle less than 0.5%) and synthetic cannabinoid 5F-APINACA (4.1%) (graphic in LC-MS/MS) associated with methadone (1.0%) (graphic in GC/MS).

In the second seizure (Case#2, Figure 3), there were three different objects: two of them were long, thin bars of hashish containing 11.85% and 10.75% Δ^9^THC concentrations (Rep A and Rep B), respectively; the third one resembled small fat cigars with a brown vegetal resinous substance (Rep C) containing Δ9THC = 0.27% (level of active principle less than 0.5% the threshold for psychoactive effects) and the synthetic cannabinoid 5F-APINACA = 4.1%, associated with methadone = 1.0%.

The presence in the same seizure of cannabis, NPSs, and traditional illicit drugs is worrying evidence, considering that users could be unaware of taking Δ^9^THC, NPSs, or other drugs.

## 4. Discussion

The COVID-19 pandemic has resulted in the widespread implementation of containment measures throughout the majority of the countries, such as lockdowns, travel restrictions, and the closing of non-essential businesses [18]. The introduction of these measures has had a major impact in all aspects of life and particularly on our economy. Much like legal businesses, drug trafficking has been affected by the COVID-19 pandemic.

Several studies have been conducted in different countries to analyze the economic trend of international drug trafficking, in order to evaluate the impact of the pandemic. The COVID-19 pandemic has consistently raised the number of drug seizures, in the USA as in Europe. A decrease in the first months of the pandemic was reported, followed by a remarkable increase where the number of drug seizures exceeded the highest pre-COVID-19 count [19]. The same trend has been observed by the FTL, both in quantitative and qualitative ways, comparing pre-pandemic case history with the ongoing pandemic period. The analysis of drug seizures occurring in Naples through the pre- and ongoing-pandemic years (2018–first semester of 2021) showed a decrease of seizures during the first wave of the pandemic followed by a huge increase in the remaining months of 2020 that exceeded the values of the pre-COVID-19 period. The first semester of 2021 showed also a remarkable increase of the number of seizures (65 in total) compared to the pre-pandemic period, with only 61 cases reported in 2019. A similar trend has been observed in other countries: in the USA, a decrease in the number of seizures from March 2020 through April 2020 was recorded, with a significant increase starting from August/September 2020, exceeding the highest pre-COVID-19 count [19]. According to the EMCDDA reports [20], seizures of illegal drugs in some EU countries during the first half of 2020 have been higher than those in the same months of previous years.

Although drug seizures are an unknown and very small fraction of drug production rates in the world [1,21], the increased number of seizures can be considered a potential indicator of the increased drug demand observed during the COVID-19 pandemic. During the pandemic, drug demand and drug supply have increased globally due to the isolation of people in their domestic environments often associated with psychological distress, health-related anxiety, and fear for the near future [22]. There is enough evidence of widespread adverse mental health symptoms [23], including depression, suicidal ideation, and abuse of licit and illicit substances [24].

It is also well known that during the pandemic most countries imposed strict border restrictions for all types of transportation (terrestrial, aerial, and maritime) by setting up checkpoints in order to enforce the quarantine order [20]. At the quarantine and border checkpoints, police enforcements have paid attention not only to infected persons and violations of the lockdown but also to drug trafficking. Different strategies of drug trafficking, supply, and distribution have been adopted to overcome the restrictions due to the COVID-19 pandemic: drug dealers impersonating food-delivery workers or other key workers [25,26] or exploiting the increasing demand for supplies such as gloves, masks, and hand sanitizer in order to hide illicit drugs [27,28].

Furthermore, the increased drug demand has also resulted in a significant acceleration of the number of drug-related deaths. According to the Centers for Disease Control and Prevention (CDC), 100,306 overdose deaths occurred in the USA from April 2020 to April 2021, an increase of 28.5% from the 78,056 deaths of the previous year [29]. The high number of drug-related deaths has also been referred to being due to changes in drug constitution due to the reduction of their purity, which has been reported in many countries. [30,31].

COVID-19 pandemic seems to have modified drug trafficking not only quantitatively, but also qualitatively. In this study, seizures of hashish and marijuana containing a low level of active principle (<0.7%) were recorded during the first wave of pandemic. NPSs were detected by the FTL in two different seizures analyzed in June 2021. In both cases, toxicological results showed cannabis derivatives presenting different peculiarities: a remarkably unusual combination of substances was detected, since NPSs are usually not synthetized in combination with other types of NPSs or other drugs [32]. The COVID-19 pandemic seems to have changed the typology of seizures from “more traditional” drugs to others, such as NPSs, which had never been found in specific geographical areas. In Italy, during the semester following the first lockdown, there was a 200% increase of seizures of NPS, compared to the semester before the first lock-down [33].

In the past, the synthetic cannabinoid market has shown a wide range of products available from online retailers, with varying levels of concentration of plant material, designed to be smoked. Although multiple SCs have been reported in the same plant material, a single synthetic cannabinoid per sample was a more common occurrence. The combination of other drugs with SCs, although uncommon, has also been observed. SCs have previously been detected in combination with cathinones, hallucinogenics, tryptamines and phenethylamines, and opioid receptor agonists [34]. The analyses showed the combination of cannabis (0.57% THC—low level of active principle) with the synthetic cannabinoid AB-FUBINACA in one case, and an inactive cannabis preparation with the synthetic cannabinoid 5F-APINACA, associated with methadone in the other case. It should be noted that the detection of THC combined with other substances has to be considered quite unusual. According to Peck et al. (2019), most of the THC samples (98%) coming from seizures contain no impurity. However, when adulterants are found, they are mostly represented by cocaine, methamphetamine, MDMA, and SCs [35].

The detection of NPSs associated with methadone in inactive cannabis preparations is to be considered alarming, since the association of unidentified substances might represent a potential health risk to unaware consumers [5]. The polydrug presence might be aimed at obtaining pharmacological effects such as the inhibition of neuronal reuptake [36], or to enhance the desired psychoactive effects compared to when drugs are individually consumed [37].

The NPS market is highly dynamic, with great variability in the substances available over time. Of the nearly 700 new substances reported in Europe (EU), approximately half are identified and reported to the EMCDDA each year [38]. Most chemical classes of NPSs can produce adverse psychiatric and medical consequences. Patients intoxicated with NPSs are also a challenge for healthcare professionals, especially those involved in emergency medical care. The long-term neuropsychiatric consequences of NPS are still not known, but acute effects (e.g., agitation, hallucinations, psychosis, violent behavior, and coma) are commonly associated with their use and abuse [5]. The growing popularity and wide availability of NPSs, combined with a lack of information on their acute toxicity and chronic harms, have raised concerns regarding the risk to public health and their social and economic impact [39]. Symptoms may resolve spontaneously, but in most of cases they range from mild to moderate intoxication, including nausea, weakness, tachycardia, hypertension, and psychosis [40,41,42]. Several reports have described users in “excited delirium”, being agitated and sweating profusely [42]. Severe symptoms include cardiac arrhythmias, myocardial infarction, hyperthermia, respiratory depression, flaccid paralysis, rhabdomyolysis, coma, and even death. Protocols for emergency responders are available for each type of symptom, with antidotes that are not based on pharmacological evidence, but on what is effective for the individual [5].

## 5. Conclusions

The COVID-19 pandemic has had a remarkable impact on the Italian drug market, raising the number of drug seizures and changing their typology. A similar trend has been observed by the FTL, comparing pre-pandemic case history with the ongoing pandemic period. In Naples (Southern Italy), cannabis, both as marijuana and hashish, remains the most-seized drug, followed by cocaine, heroin, prescribed drugs, and ketamine and amphetamine/MDMA, but synthetic cannabinoids (AB-FUBINACA and 5F-APINACA) appeared for the first time during COVID-19 pandemic.

COVID-19 pandemic seems to have modified drug trafficking not only quantitatively, according to the increased drug demand, but also qualitatively. Changes in drug constitution may have unpredictable consequences due to the reduction in their purity. The detection of an NPS associated with methadone on inactive cannabis preparations (Δ^9^THC = 0.27%) is alarming evidence, that requires further research studies. Users can be unaware of taking NPSs or other drugs, in association with a derivative of cannabis that they consider of “natural” origin. Therefore, according to the international warning for the intake of contaminated cannabis preparations by unsuspecting consumers, intoxication cases can be unpredictable in the near future. Specific analytic NPS identifying methods are essential in routine toxicology investigation, on biological and autoptic materials, in order to provide a correct diagnosis both in cases of clinical emergency and post-mortem.

## Figures and Tables

**Table 1:** The illicit drugs detected from seizures in Naples (Italy) from January 2018 to June 2021.

YEAR	Cannabis		Cocaine	Heroin	Ketamine	Amphetamines MDMA	NPS	Prescribed Drugs		TOTAL
	Hashish (THC < 0.7%)	Marihuana (THC < 0.7%)						Methadone	Prazepam	
**2018**	13	15 (1)	15	7	1	2	. .	. .	. .	**53**
**2019**	20	16 (1)	22	2	1	. .	. .	. .	. .	**61**
**2020**	21 (1)	34 (3)	26	6	. .	. .	. .	1	1	**89**
**2021 1° semester**	24 (2)	14 (2)	18	5	. .	. .	2	2	. .	**65**
**Total** **2018–2021**	**157**		**81**	**20**	**2**	**2**	**2**	**4**		**268**

## Data Availability

Not applicable.
